# Supplementary material for: Dynamic blebbing and absence of organelle transfer during mouse oocyte formation
Source: EMBO J. 2026 Apr 21;45(11):3880–925. doi: 10.1038/s44318-026-00780-6 (PMC13226715; doi:10.1038/s44318-026-00780-6)
Supplement: Supplementary file 1 — Appendix [file 44318_2026_780_MOESM1_ESM.pdf]

Appendix for

Dynamic blebbing and absence of organelle transfer during mouse oocyte formation

Table of contents:

		Page
Appendix Table S1:	List of genotyping primers	2
Appendix Table S2:	List of RT-qPCR primers	3

Appendix Table S1: List of genotyping primers

Name	Sequence (5' to 3')
SRY-2	TCT TAA ACT CTG AAG AAG AGA C
SRY-4	GTC TTG CCT GTA TGT GAT GG
Xist-14	GTA GAT ATG GCT GTT GTC AC
Xist-16	CTC CAT CCA AGT TCT TTC TG
P1	TCC CTC GTG ATC TGC AAC TCC AGT C
P2	AAC CCC AGA TGA CTA CCT ATC CTC C
P3	GCT GCA GGT CGA GGG ACC
P4	ATC GAG CTG CGT ATG GAA GG
P5	GTC TGG CAA CTG GAC AAC CT
P6	GCT CAG TTG GGC TGT TTT GG
P7	CCT TCC ATA CGC AGC TCG AT

Appendix Table S2: List of RT-qPCR primers

Gene symbol	Forward (5′ to 3′)	Reverse (5′ to 3′)
Dnmt3a	GAC TCG CGT GCA ATA ACC TTA G	GGT CAC TTT CCC TCA CTC TGG
Dnmt3b	CTC GCA AGG TGT GGG CTT TTG TAA C	CTG GGC ATC TGT CAT CTT TGC ACC
Figla	ACA GAG CAG GAA GCC CAG TA	TGG GTA GCA TTT CCC AAG AG
Hormad1	GGC TCC TAG CTG TTT CAG TAT CT	GCA TCC ACT TCA CTA GCT GTG
Nobox	CGT TCC TGG CAG TGA CAG CAT A	GGA ATG AAC CCA ACT GGC TGC T
Rec8	TAT GTG CTG GTA AGA GTG CAA C	TGT CTT CCA CAA GGT ACT GGC
Rplp0	CAA AGC TGA AGC AAA GGA AGA G	AAT TAG ACA GGC GTG ACT TGG TTG
Smc1b	GAG AAT TTC AAG TCG TGG CGA	CAG GTT TTC CAG TAT GTG CTC C
Sohlh1	GCC AAA CCA TCT GCT GTG TCT C	AAG GTC TCT CCA GCA GCT CTG A
Spo11	GAA GTG CCT GCC TTC ACA AT	GCC GAC AGA ATC ATC AAA CAT
Zp3	TCT TCA CAG TTC CAG ATC CAT G	TGG TCG TTG GCC TTT CCA AGG A
