# Supplementary material for: Dynamic blebbing and absence of organelle transfer during mouse oocyte formation
Source: EMBO J. 2026 Apr 21;45(11):3880–925. doi: 10.1038/s44318-026-00780-6 (PMC13226715; doi:10.1038/s44318-026-00780-6)
Supplement: Supplementary file 3 — Movie EV1 [file 44318_2026_780_MOESM3_ESM.zip › Movie EV1/Legend Movie EV1.docx]

**Movie EV1: Long-term live imaging of germ cell dynamics during oocyte formation (related to Figure 3A).**

Representative time-lapse imaging of an E12.5 + 4d gonad cultured *ex vivo* expressing H2B-mCherry (magenta) and stained with PlasMem Bright Green (green). The movie spans 130 h 40 min of development and shows germ cell growth and degeneration of a germ cell cluster. Time is shown as hours:minutes:seconds.
