# Supplementary material for: Dynamic blebbing and absence of organelle transfer during mouse oocyte formation
Source: EMBO J. 2026 Apr 21;45(11):3880–925. doi: 10.1038/s44318-026-00780-6 (PMC13226715; doi:10.1038/s44318-026-00780-6)
Supplement: Supplementary file 4 — Movie EV2 [file 44318_2026_780_MOESM4_ESM.zip › Movie EV2/Legend Movie EV2.docx]

**Movie EV2: Live imaging of germ cell cluster degeneration observed in a freshly isolated ovary (related to Figure 3G).**

Representative time-lapse imaging of an E16.5 ovary expressing H2B-mCherry (magenta) and stained with PlasMem Bright Green (green). The movie spans 6 h 50 min and shows synchronous degeneration of germ cells within a cluster. Time is shown as hours:minutes:seconds.
