# Supplementary material for: Dynamic blebbing and absence of organelle transfer during mouse oocyte formation
Source: EMBO J. 2026 Apr 21;45(11):3880–925. doi: 10.1038/s44318-026-00780-6 (PMC13226715; doi:10.1038/s44318-026-00780-6)
Supplement: Supplementary file 5 — Movie EV3 [file 44318_2026_780_MOESM5_ESM.zip › Movie EV3/Legend Movie EV3.docx]

**Movie EV3: Live imaging of germ cell nuclear rotation (related to Figure EV4A).**

Representative time-lapse imaging of an E12.5 + 4d gonad stained with Hoechst 33342. The movie shows rotational movement of germ cell nuclei. Time is shown as hours:minutes:seconds.
