# Supplementary material for: Dynamic blebbing and absence of organelle transfer during mouse oocyte formation
Source: EMBO J. 2026 Apr 21;45(11):3880–925. doi: 10.1038/s44318-026-00780-6 (PMC13226715; doi:10.1038/s44318-026-00780-6)
Supplement: Supplementary file 6 — Movie EV4 [file 44318_2026_780_MOESM6_ESM.zip › Movie EV4/Legend Movie EV4.docx]

**Movie EV4: Live imaging of germ cell blebbing (related to Figure 4A,B).**

Representative time-lapse imaging of an E12.5 + 2d gonad expressing Stella-ECFP (green) and stained with PlasMem Bright Red (magenta). The movie shows membrane blebbing in germ cells. Time is shown as hours:minutes:seconds.
