# Supplementary material for: Dynamic blebbing and absence of organelle transfer during mouse oocyte formation
Source: EMBO J. 2026 Apr 21;45(11):3880–925. doi: 10.1038/s44318-026-00780-6 (PMC13226715; doi:10.1038/s44318-026-00780-6)
Supplement: Supplementary file 7 — Movie EV5 [file 44318_2026_780_MOESM7_ESM.zip › Movie EV5/Legend Movie EV5.docx]

**Movie EV5: Live imaging of germ cells during cytokinesis (related to Figure EV5B).**

Representative time-lapse imaging of an E12.5 + 2d gonad expressing Stella-ECFP (green) and stained with PlasMem Bright Red (magenta), showing a germ cell undergoing cytokinesis. Time is shown as hours:minutes:seconds.
