# Supplementary material for: Dynamic blebbing and absence of organelle transfer during mouse oocyte formation
Source: EMBO J. 2026 Apr 21;45(11):3880–925. doi: 10.1038/s44318-026-00780-6 (PMC13226715; doi:10.1038/s44318-026-00780-6)
Supplement: Supplementary file 8 — Movie EV6 [file 44318_2026_780_MOESM8_ESM.zip › Movie EV6/Legend Movie EV6.docx]

**Movie EV6: Live imaging of actin dynamics during germ cell blebbing (related to Figure EV5C).**

Representative time-lapse imaging of an E12.5 + 1d gonad stained with PlasMem Bright Red (left) and SiR-Actin (center) and expressing Stella-ECFP (right). Time is shown as hours:minutes:seconds.
