# Supplementary material for: Dynamic blebbing and absence of organelle transfer during mouse oocyte formation
Source: EMBO J. 2026 Apr 21;45(11):3880–925. doi: 10.1038/s44318-026-00780-6 (PMC13226715; doi:10.1038/s44318-026-00780-6)
Supplement: Supplementary file 9 — Movie EV7 [file 44318_2026_780_MOESM9_ESM.zip › Movie EV7/Legend Movie EV7.docx]

**Movie EV7: Effects of BMP and RA signaling inhibition on germ cell blebbing (related to Figures 4I and EV6A).**

Representative time-lapse imaging of E11.5 + 3d gonads expressing Stella-ECFP (green) and stained with PlasMem Bright Red (magenta). DMSO-treated control is shown on the left, LDN193189-treated sample in the center, and BMS493-treated sample on the right. Time is shown as hours:minutes:seconds.
