# Supplementary material for: Dynamic blebbing and absence of organelle transfer during mouse oocyte formation
Source: EMBO J. 2026 Apr 21;45(11):3880–925. doi: 10.1038/s44318-026-00780-6 (PMC13226715; doi:10.1038/s44318-026-00780-6)
Supplement: Supplementary file 10 — Movie EV8 [file 44318_2026_780_MOESM10_ESM.zip › Movie EV8/Legend Movie EV8.docx]

**Movie EV8: Live imaging of Golgi dynamics during oocyte formation (related to Figure EV7A).**

Representative time-lapse imaging of an E15.5 + 2d ovary expressing Golgi-EGFP (green) and stained with PlasMem Bright Red (magenta). Time is shown as hours:minutes:seconds.
