# Supplementary material for: Dynamic blebbing and absence of organelle transfer during mouse oocyte formation
Source: EMBO J. 2026 Apr 21;45(11):3880–925. doi: 10.1038/s44318-026-00780-6 (PMC13226715; doi:10.1038/s44318-026-00780-6)
Supplement: Supplementary file 12 — Movie EV10 [file 44318_2026_780_MOESM12_ESM.zip › Movie EV10/Legend Movie EV10.docx]

**Movie EV10: Live imaging of photoconverted mitochondria during oocyte formation (related to Figure 5C).**

Representative time-lapse imaging of an E12.5 + 6d gonad expressing Mito-Dendra2 and stained with PlasMem Bright Green (green). Green Mito-Dendra2 is shown in green and photoconverted Red Mito-Dendra2 in magenta. Time is shown as hours:minutes:seconds.
