# Supplementary material for: Dynamic blebbing and absence of organelle transfer during mouse oocyte formation
Source: EMBO J. 2026 Apr 21;45(11):3880–925. doi: 10.1038/s44318-026-00780-6 (PMC13226715; doi:10.1038/s44318-026-00780-6)
Supplement: Supplementary file 13 — Movie EV11 [file 44318_2026_780_MOESM13_ESM.zip › Movie EV11/Legend Movie EV11.docx]

**Movie EV11: Live imaging of centrosome dynamics during oocyte formation (related to Figure EV9C).**

Representative time-lapse imaging of an E12.5 + 7d gonad expressing EGFP-CETN2 (green) and stained with PlasMem Bright Red (magenta). Time is shown as hours:minutes:seconds.
