# Supplementary material for: Dynamic blebbing and absence of organelle transfer during mouse oocyte formation
Source: EMBO J. 2026 Apr 21;45(11):3880–925. doi: 10.1038/s44318-026-00780-6 (PMC13226715; doi:10.1038/s44318-026-00780-6)
Supplement: Supplementary file 14 — Movie EV12 [file 44318_2026_780_MOESM14_ESM.zip › Movie EV12/Legend Movie EV12.docx]

**Movie EV12: Live imaging of photoconverted centrosomes during oocyte formation (related to Figures 6F and EV10B).**

Representative time-lapse imaging of an E12.5 + 5d gonad expressing mKikGR-CETN2 and stained with PlasMem Bright Green. Left: live imaging showing PlasMem Bright Green (green), Green mKikGR-CETN2 (green), and photoconverted Red mKikGR-CETN2 (magenta). Right: 3D reconstruction of the targeted germ cell, with the cell membrane shown in green and the photoconverted Red mKikGR-CETN2 focus shown in magenta. Time is shown as hours:minutes:seconds.
