# Supplementary figures and images for: Dynamic blebbing and absence of organelle transfer during mouse oocyte formation
Source: EMBO J. 2026 Apr 21;45(11):3880–925. doi: 10.1038/s44318-026-00780-6 (PMC13226715; doi:10.1038/s44318-026-00780-6)

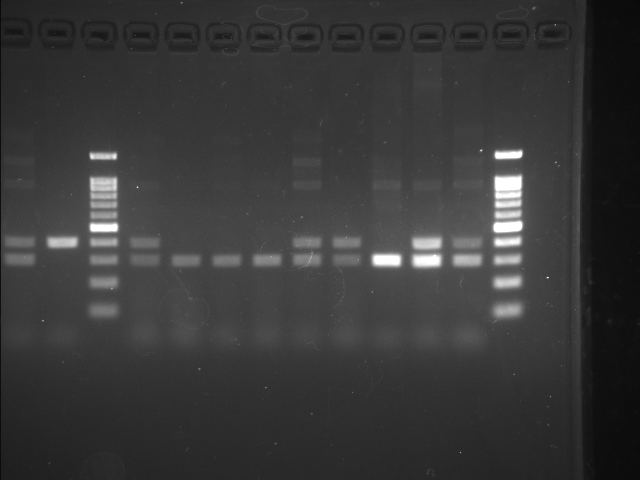

Supplement: Supplementary file 24 — Figure EV5 Source Data [file 44318_2026_780_MOESM24_ESM.zip › Figure_EV5/EV5A/SourceData_FigEV5A.TIF]
