# Supplementary material for: Dynamic blebbing and absence of organelle transfer during mouse oocyte formation
Source: EMBO J. 2026 Apr 21;45(11):3880–925. doi: 10.1038/s44318-026-00780-6 (PMC13226715; doi:10.1038/s44318-026-00780-6)
Supplement: Supplementary file 29 — Expanded View Figures [file 44318_2026_780_MOESM29_ESM.pdf]

Expanded View Figures

Figure EV1. Three-dimensional reconstruction and germ cell identification during ex vivo culture.

(**A**) Three-dimensional reconstructed images of cells in E12.5 gonads expressing Stella-ECFP during an 8-day ex vivo culture. Gonads were stained with Hoechst 33342 and PlasMem Bright Red. Germ cell membranes (green), germ cell nuclei (magenta), somatic cell membranes (cyan), and somatic cell nuclei (yellow) are shown. Confocal z-sections were acquired at 1 µm intervals, and 3D images were reconstructed orthogonally. Identical images of E12.5 + 2 d, E12.5 + 5 d, and E12.5 + 8 d gonads are shown in Fig. 2B. Scale bar, 10 µm. (**B**) Development of germ cells in E12.5 gonads expressing Stella-ECFP during an 8-day ex vivo culture. Gonads were stained with PlasMem Bright Red. The merged image shows Stella-ECFP expression (green) alongside the PlasMem signal (magenta). Stella-ECFP expression weakened around day 5 of culture but strengthened again by days 7 and 8. Asterisks indicate cells with weakened Stella-ECFP signals. Scale bar, 10 µm.

 © The Author(s)

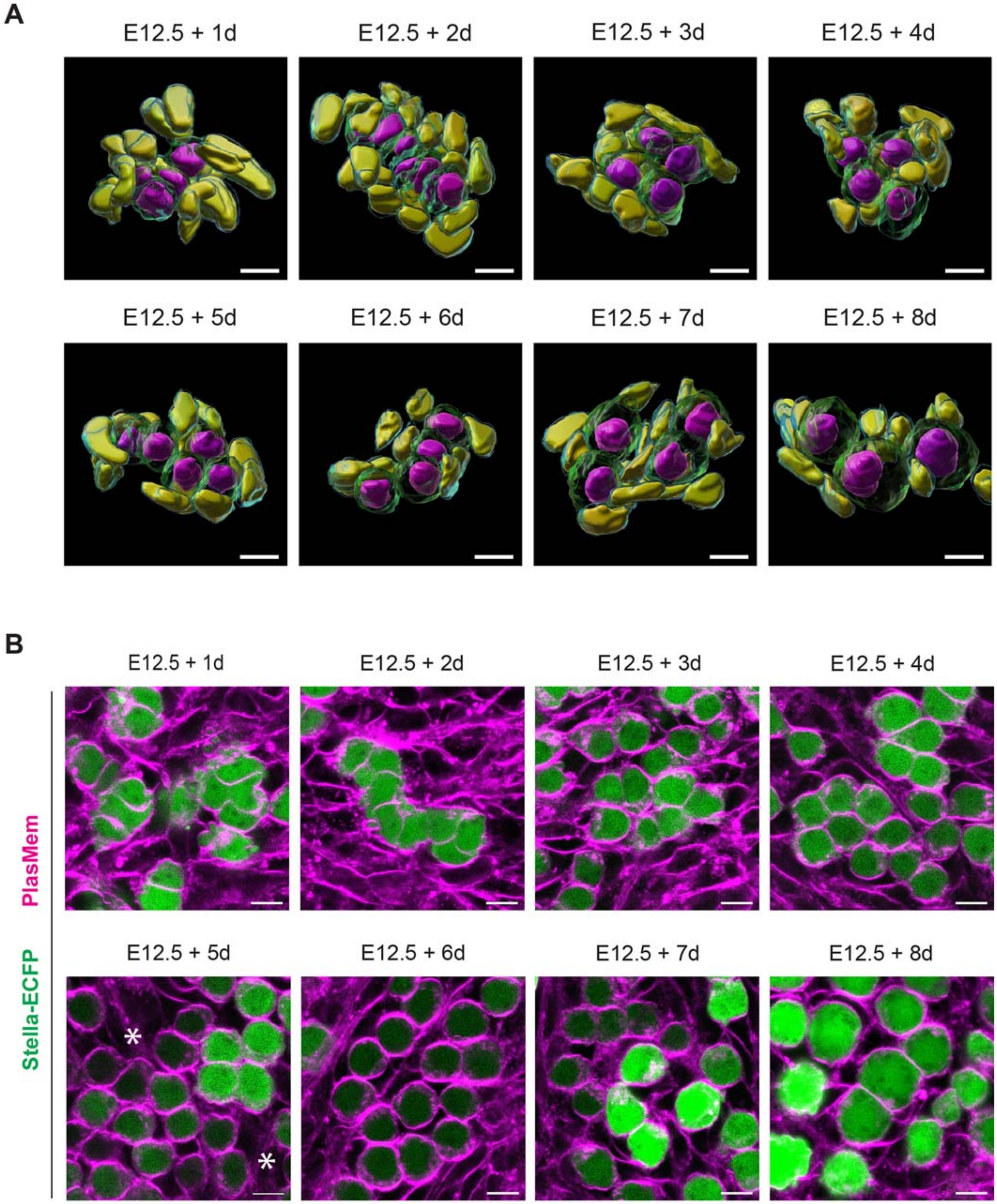
A
E12.5 + 1d
E12.5 + 2d
E12.5 + 3d
E12.5 + 4d
E12.5 + 5d
E12.5 + 6d
E12.5 + 7d
E12.5 + 8d
B
E12.5 + 1d
E12.5 + 2d
E12.5 + 3d
E12.5 + 4d
PlasMem
Stella-ECFP
E12.5 + 5d
E12.5 + 6d
E12.5 + 7d
E12.5 + 8d

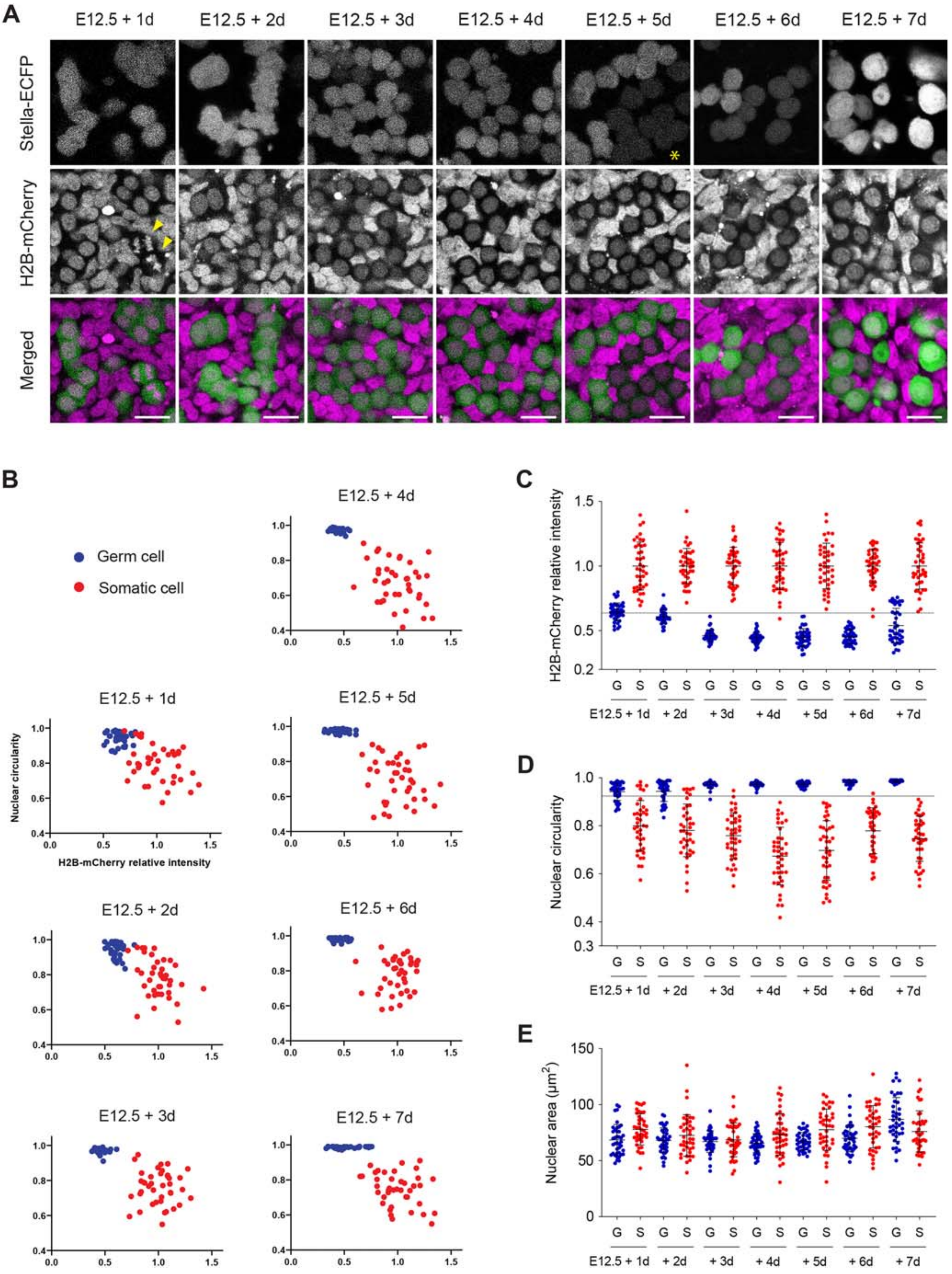
A
E12.5 + 1d
E12.5 + 2d
E12.5 + 3d
E12.5 + 4d
E12.5 + 5d
E12.5 + 6d
E12.5 + 7d
Stella-ECFP
H2B-mCherry
Merged
B
Germ cell
Somatic cell
Nuclear circularity
H2B-mCherry relative intensity
C
H2B-mCherry relative intensity
D
Nuclear circularity
E
Nuclear area (μm²)
G S G S G S G S G S G S G S
E12.5 + 1d
+ 2d
+ 3d
+ 4d
+ 5d
+ 6d
+ 7d

 © The Author(s)

◀ **Figure EV2. Validation of germ and somatic cell classification using nuclear features.**

(**A**) Development of germ cells in E12.5 gonads co-expressing Stella-ECFP and H2B-mCherry during a 7-day ex vivo culture. The merged image (bottom) shows Stella-ECFP (green) and H2B-mCherry (magenta) expression. Stella-ECFP signals weakened around day 5 of culture but strengthened by day 7. An asterisk indicates cells with weakened Stella-ECFP signals. Arrowheads mark Stella-ECFP-positive germ cells undergoing presumed anaphase chromosome segregation. Scale bar, 20 μm.
(**B**) Quantitative analysis of histone intensity and nuclear circularity in cells from E12.5 gonads co-expressing Stella-ECFP and H2B-mCherry during the 7-day ex vivo culture. From E12.5 + 3 d to E12.5 + 7 d, two distinct populations were identified: germ cells with low H2B-mCherry intensity and high nuclear circularity, and somatic cells with high H2B-mCherry intensity and low nuclear circularity. The mean H2B-mCherry intensity of somatic cells was normalized to a relative intensity of 1. (**C–E**) Quantitative analysis of histone intensity (**C**), nuclear circularity (**D**) and nuclear area (**E**) in cells from E12.5 gonads co-expressing Stella-ECFP and H2B-mCherry during the 7-day ex vivo culture. Bars represent mean values ± standard deviations. (**C**) The mean H2B-mCherry intensity of somatic cells was normalized to a relative intensity of 1. A dashed line indicates a H2B-mCherry relative intensity threshold of 0.63, distinguishing somatic cells (> 0.63; 98.8% (158/160)) and germ cells (≤ 0.63; 100% (160/160)) between E12.5 + 3 d and E12.5 + 6 d. (**D**) A dashed line indicates a nuclear circularity threshold of 0.93, distinguishing somatic cells (> 0.93; 99.0% (198/200)) and germ cells (≤ 0.93; 99.5% (199/200)) between E12.5 + 3 d and E12.5 + 7 d. (**B–E**) Germ cells (blue) and somatic cells (red) were distinguished based on Stella-ECFP expression intensity. Quantification included 40 germ cells and 40 somatic cells at each developmental time point. G germ cell, S somatic cell. Source data are available online for this figure.

© The Author(s)

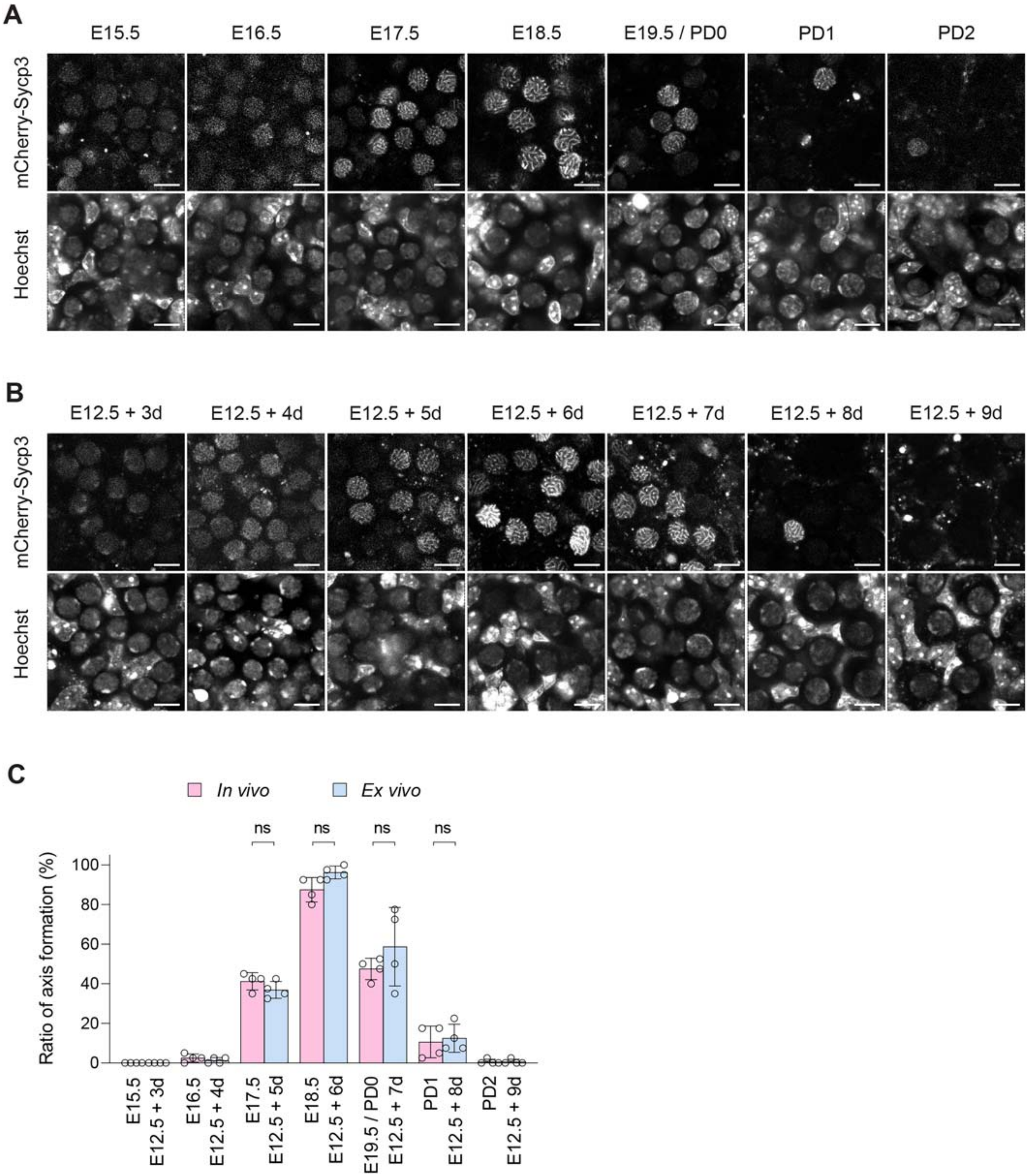
A
E15.5
E16.5
E17.5
E18.5
E19.5 / PD0
PD1
PD2
mCherry-Sycp3
Hoechst
B
E12.5 + 3d
E12.5 + 4d
E12.5 + 5d
E12.5 + 6d
E12.5 + 7d
E12.5 + 8d
E12.5 + 9d
mCherry-Sycp3
Hoechst
C
In vivo
Ex vivo
ns
ns
ns
ns
Ratio of axis formation (%)
100
80
60
40
20
0
E15.5
E12.5 + 3d
E16.5
E12.5 + 4d
E17.5
E12.5 + 5d
E18.5
E12.5 + 6d
E19.5 / PD0
E12.5 + 7d
PD1
E12.5 + 8d
PD2
E12.5 + 9d

 © The Author(s)

Figure EV3. Comparison of chromosome axis formation in germ cells during in vivo and ex vivo development.

(**A**) Representative images of germ cells in ovaries collected from E15.5 fetuses to PD2 neonates. Ovaries expressing mCherry-SYCP3 were stained with Hoechst 33342 prior to imaging. Chromosome axis formation was prominently observed between E17.5 and E19.5/PD0. Scale bar, 10 µm. (**B**) Representative images of germ cells in E12.5 gonads during a 9-day ex vivo culture. E12.5 gonads expressing mCherry-SYCP3 were stained with Hoechst 33342 prior to imaging. Chromosome axis formation was prominently observed between E12.5 + 5 d and E12.5 + 7 d. Scale bar, 10 µm. (**C**) Quantitative analysis of chromosome axis formation ratios in germ cells from ovaries and E12.5 gonads cultured ex vivo, both expressing mCherry-SYCP3. Germ cells with or without distinct axis formation were evaluated based on mCherry-SYCP3 signals from confocal z-section images. Bars represent mean values ± standard deviations. $N = 4$ gonads/ovaries; 40 germ cells per gonad/ovary. Statistical analysis was performed using a *t* test with Welch's correction. ns non-significant. Source data are available online for this figure.

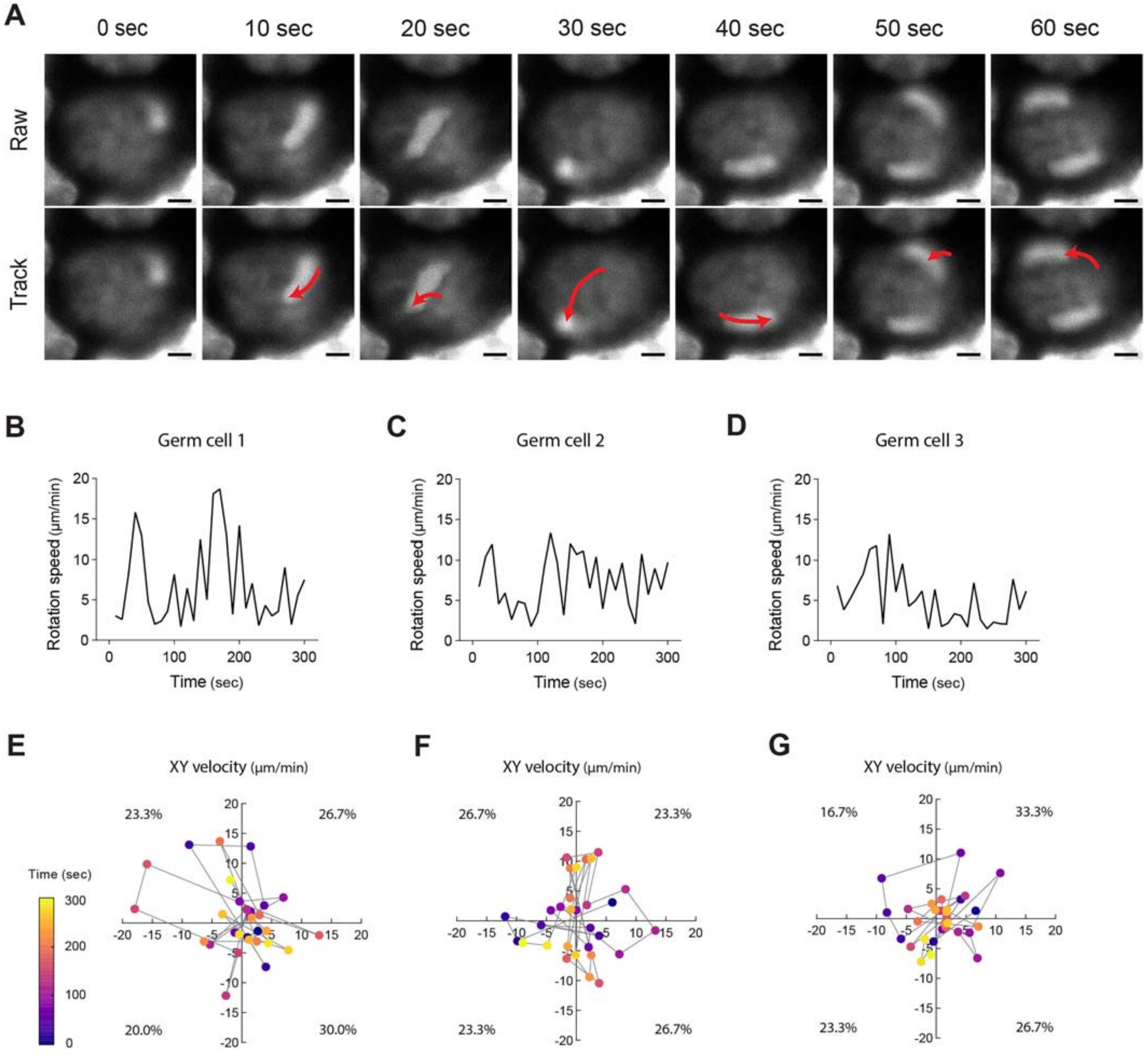

Figure EV4. Characterization of nuclear rotation dynamics in germ cells.

(**A**) Representative live imaging of a germ cell nucleus in an E12.5 + 4 d gonad cultured ex vivo. The gonad was stained with Hoechst 33342 and imaged at 10-second intervals. Raw and tracking images are shown, with red lines indicating trajectories of nuclear rotation. Scale bar, 2 μm. See also Movie EV3. (**B–D**) Time-lapse quantification of nuclear rotation speed for three representative germ cells. Rotation speed was measured every 10 s over a 300-second period. (**E–G**) Plots of X velocity (*x* axis) versus Y velocity (*y* axis) recorded at 10-s intervals for a 300-second sequence. Each plot corresponds to the germ cells analyzed in (**B–D**), respectively. The number within each quadrant indicates the proportion of XY velocity plots among the 30 measurements. Source data are available online for this figure.

 © The Author(s)

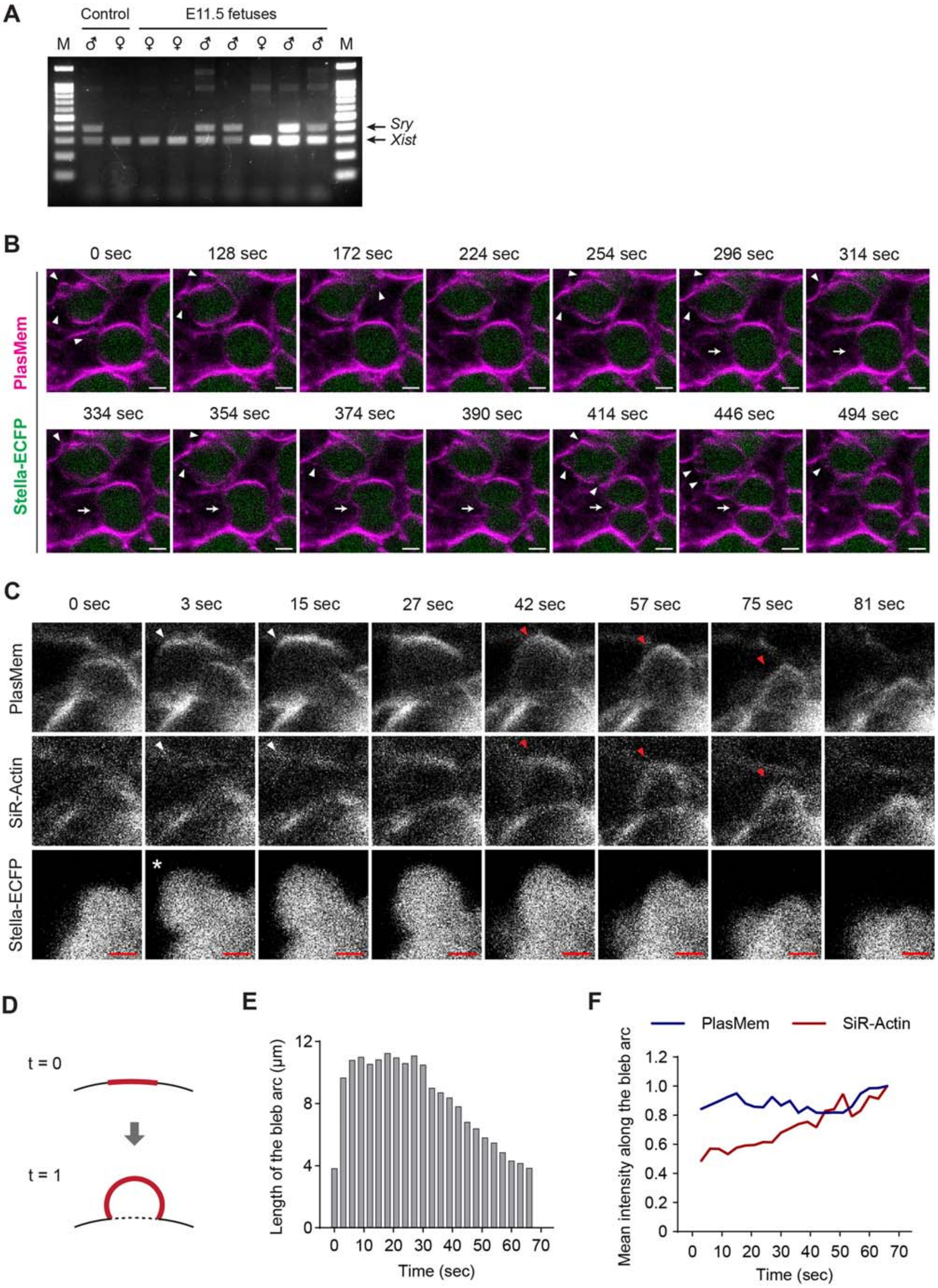
A
Control
E11.5 fetuses
M ♂ ♀ ♀ ♀ ♂ ♂ ♀ ♂ ♂ M
Sry
Xist
B
0 sec
128 sec
172 sec
224 sec
254 sec
296 sec
314 sec
334 sec
354 sec
374 sec
390 sec
414 sec
446 sec
494 sec
PlasMem
Stella-ECFP
C
0 sec
3 sec
15 sec
27 sec
42 sec
57 sec
75 sec
81 sec
PlasMem
SiR-Actin
Stella-ECFP
D
t = 0
t = 1
E
Length of the bleb arc (µm)
Time (sec)
F
PlasMem
SiR-Actin
Mean intensity along the bleb arc
Time (sec)

Figure EV5. Validation of germ cell blebbing and analysis of actin dynamics.

(**A**) PCR analysis of E11.5 fetuses using *Sry* and *Xist* primers to determine fetal sex. M marker. (**B**) Live imaging of germ cells in an E12.5 gonad expressing Stella-ECFP after 2 days of ex vivo culture. The gonad was stained with PlasMem Bright Red and imaged every 2 s. Stella-ECFP (green) and PlasMem (red) signals are shown as merged images. Arrowheads mark blebs, and arrows indicate the position of cytokinesis. Scale bar, 5 µm. See also Movie EV5. (**C**) Representative live-imaging of blebbing in an E12.5 gonad expressing Stella-ECFP after 1 day of ex vivo culture. The gonad was stained with PlasMem Bright Red and SiR-Actin. Images were captured every 3 s. An asterisk marks an emerging bleb. White arrowheads indicate cell membranes with weak SiR-Actin signals, while red arrowheads denote membranes with distinct SiR-Actin signals. Scale bar, 2 µm. See also Movie EV6. (**D–F**) Quantitative analysis of the blebbing shown in (**C**). (**D**) Schematic illustration of the measurement. The red line indicates the bleb arc, representing the measured linear region of interest. (**E**) Quantification of the bleb arc length over time. The arc length decreases after approximately 30 s. (**F**) Mean intensities of PlasMem Bright Red (blue) and SiR-Actin (red) along the bleb arc over time. PlasMem intensity remained stable, while SiR-Actin intensity increased as the bleb shrank. Source data are available online for this figure.

 © The Author(s)

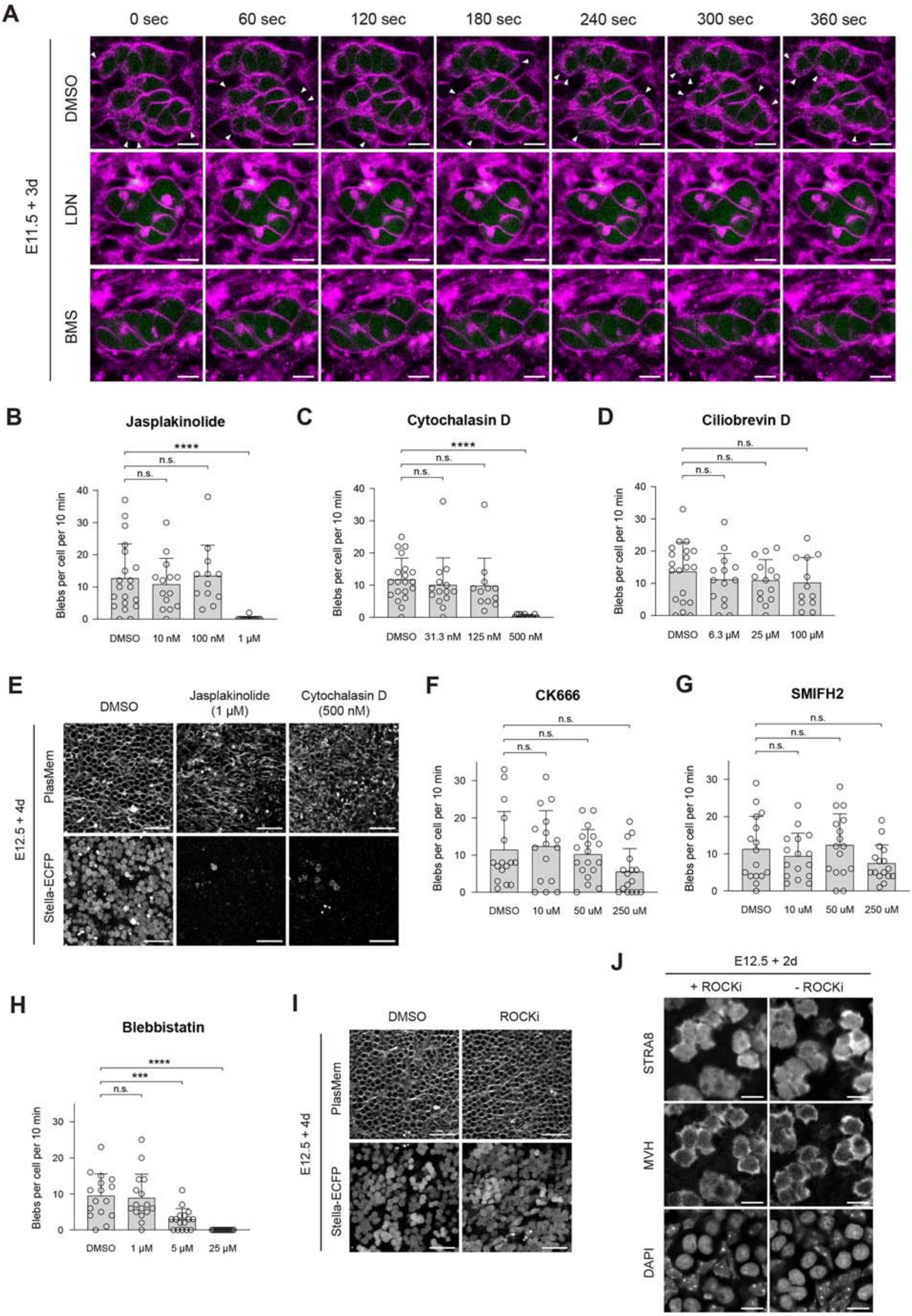

A
0 sec
60 sec
120 sec
180 sec
240 sec
300 sec
360 sec
E11.5 + 3d
DMSO
LDN
BMS
B
Jasplakinolide
Blebs per cell per 10 min
DMSO
10 nM
100 nM
1 μM
C
Cytochalasin D
DMSO
31.3 nM
125 nM
500 nM
D
Ciliobrevin D
DMSO
6.3 μM
25 μM
100 μM
E
DMSO
Jasplakinolide (1 μM)
Cytochalasin D (500 nM)
E12.5 + 4d
PlasMem
Stella-ECFP
F
CK666
DMSO
10 uM
50 uM
250 uM
G
SMIFH2
DMSO
10 uM
50 uM
250 uM
H
Blebbistatin
DMSO
1 μM
5 μM
25 μM
I
DMSO
ROCKi
E12.5 + 4d
PlasMem
Stella-ECFP
J
E12.5 + 2d
+ ROCKi
- ROCKi
STRA8
MVH
DAPI

Figure EV6. Analysis of signaling pathways regulating blebbing in germ cells.

(**A**) Representative live imaging of E11.5 + 3 d gonads under inhibition of meiotic initiation signals. Female E11.5 gonads were cultured for 3 consecutive days with DMSO, LDN193189 (500 nM) or BMS493 (10 μM) treatments, followed by staining with PlasMem Bright Red. Arrowheads indicate blebs. Scale bar, 10 μm. See also Movie EV7. (**B–D**) Blebbing frequency following treatment with Jasplakinolide (**B**), Cytochalasin D (**C**), or Ciliobrevin D (**D**). The number of blebs was counted from live imaging of E12.5 + 2 d gonads expressing Stella-ECFP. Each inhibitor was supplemented 16 h prior to imaging. Bars represent mean values + standard deviations. Statistical analysis was performed using a *t* test with Welch's correction. Sample sizes: (**B**) DMSO ($n = 20$), 10 nM ($n = 14$), 100 nM ($n = 13$), 1 μM ($n = 14$); (**C**) DMSO ($n = 20$), 31.3 nM ($n = 14$), 125 nM ($n = 12$), 500 nM ($n = 12$); (**D**) DMSO ($n = 20$), 6.3 μM ($n = 14$), 25 μM ($n = 14$), 100 μM ($n = 12$). ns, non-significant; ****$P = 0.000048$ (Jasplakinolide); ****$P = 0.00000029$ (Cytochalasin D). (**E**) Representative images of cells in E12.5 + 4 d gonads expressing Stella-ECFP. Gonads were cultured with DMSO, Jasplakinolide (1 μM), or Cytochalasin D (500 nM) for 16 h between E12.5 + 1 d and E12.5 + 2 d, followed by staining with PlasMem Bright Red. Stella-ECFP-positive cells were abundant in samples treated with DMSO but were scarcely observed following treatment with Jasplakinolide or Cytochalasin D. Scale bar, 50 μm. (**F–H**) Blebbing frequency following treatment CK666 (**F**), SMIFH2 (**G**), or Blebbistatin (**H**). The number of blebs was counted from live imaging of E12.5 + 2 d gonads expressing Stella-ECFP. Each inhibitor was supplemented 16 h prior to imaging. Bars represent mean values + standard deviations. Statistical analysis was performed using a *t* test with Welch's correction. Sample sizes: (**F**) DMSO ($n = 16$), 10 μM ($n = 16$), 50 μM ($n = 18$), 250 μM ($n = 16$); (**G**) DMSO ($n = 16$), 10 μM ($n = 16$), 50 μM ($n = 16$), 250 μM ($n = 16$); (**H**) DMSO ($n = 16$), 1 μM ($n = 16$), 5 μM ($n = 16$), 25 μM ($n = 16$). ns, non-significant; ***$P = 0.00092$; ****$P = 0.000017$. (**I**) Representative images of cells in E12.5 + 4 d gonads expressing Stella-ECFP. Gonads were cultured with either DMSO or ROCKi (12 nM) for 16 h between E12.5 + 1 d and E12.5 + 2 d, followed by staining with PlasMem Bright Red. Scale bar, 50 μm. (**J**) Representative immunostaining of STRA8 in cultured gonads following ROCKi treatment. E12.5 + 2 d gonads were stained with antibodies against STRA8 and MVH, followed by DAPI counterstaining. STRA8 signals were detected in germ cells irrespective of ROCKi treatment. Scale bar, 10 μm. Source data are available online for this figure.

 © The Author(s)

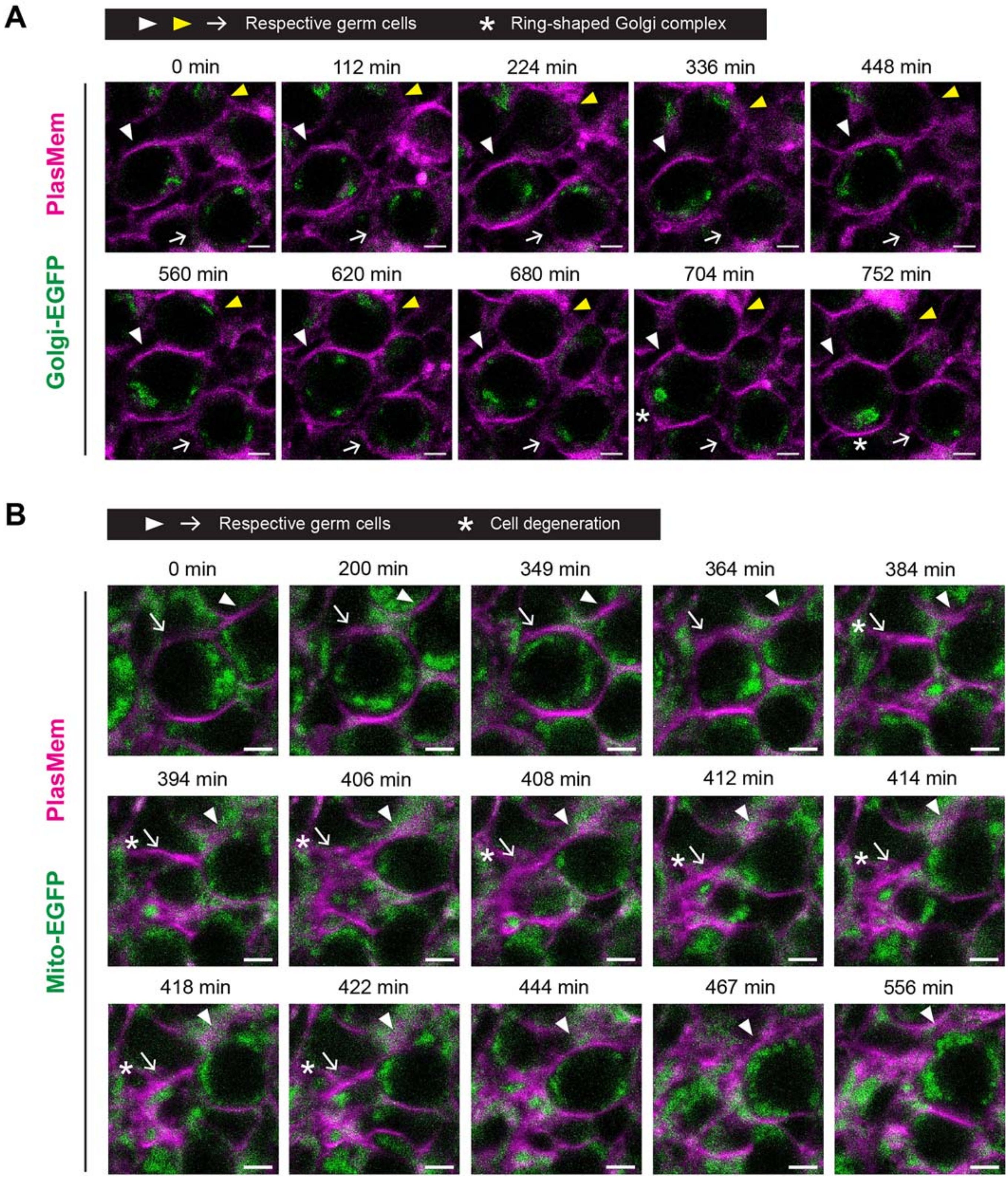
A
Respective germ cells
Ring-shaped Golgi complex
0 min
112 min
224 min
336 min
448 min
560 min
620 min
680 min
704 min
752 min
PlasMem
Golgi-EGFP
B
Respective germ cells
Cell degeneration
0 min
200 min
349 min
364 min
384 min
394 min
406 min
408 min
412 min
414 min
418 min
422 min
444 min
467 min
556 min
PlasMem
Mito-EGFP

Figure EV7. Live imaging of Golgi and mitochondrial dynamics during oocyte formation.

(**A**) Representative live imaging of an E15.5 + 2 d ovary expressing Golgi-EGFP. The ovary was stained with PlasMem Bright Red and imaged at 4-min intervals. Golgi-EGFP (green) and PlasMem (magenta) signals are shown as merged images. Tracked germ cells are indicated by arrows, white arrowheads, and yellow arrowheads, respectively. Asterisks mark ring-shaped Golgi complexes. Scale bar, 5 µm. See also Movie EV8. (**B**) Representative live imaging of an E14.5 + 6 d ovary expressing Mito-EGFP. The ovary was stained with PlasMem Bright Red and imaged at 1-min intervals. Merged images show Mito-EGFP (green) and PlasMem (magenta) signals. Arrows and arrowheads indicate a tracked germ cell and its adjacent germ cell, respectively. Asterisks denote the degeneration of the tracked germ cell. Scale bar, 5 µm. See also Movie EV9.

 © The Author(s)

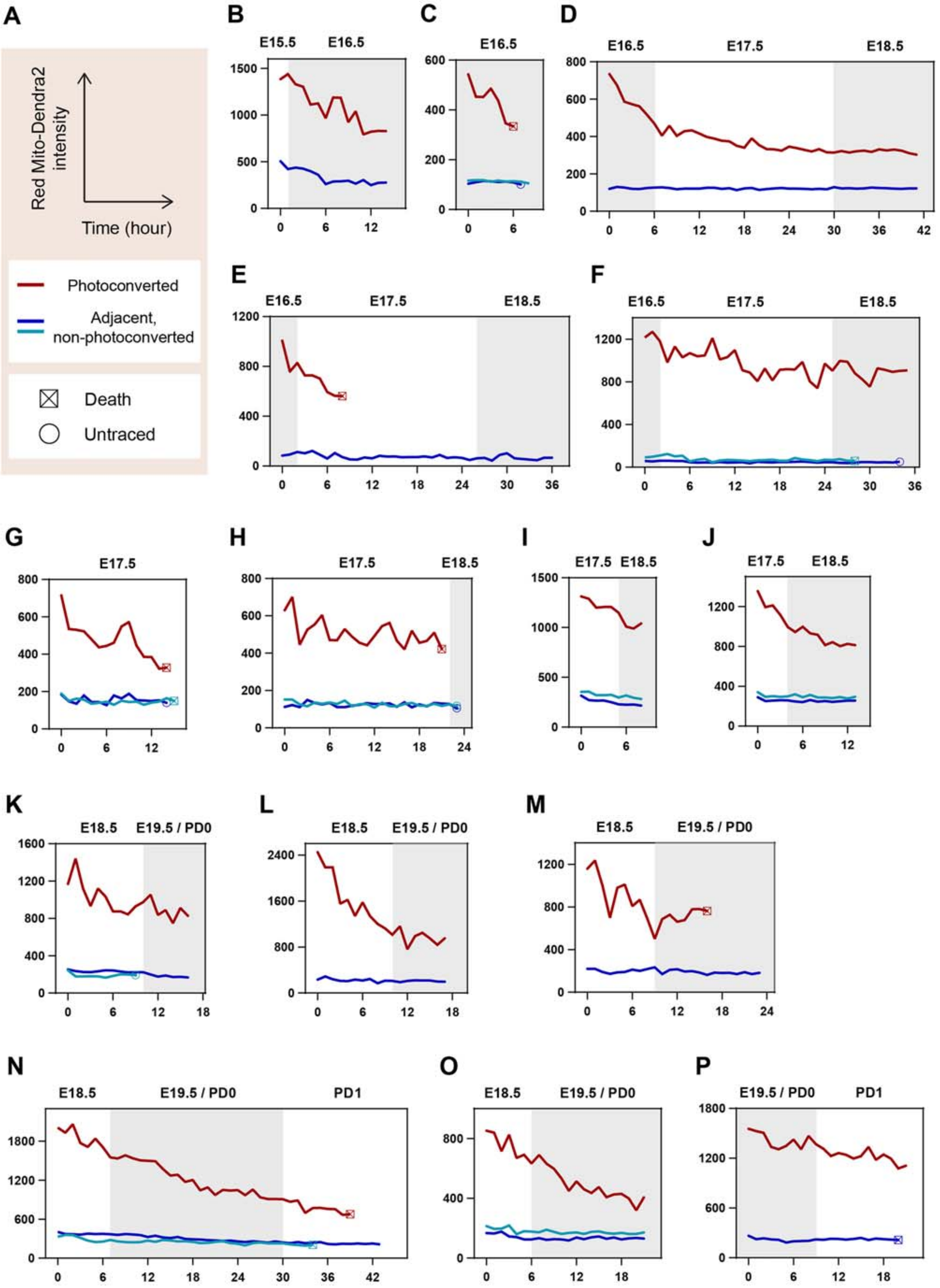
A
Red Mito-Dendra2 intensity
Time (hour)
Photoconverted
Adjacent, non-photoconverted
Death
Untraced
B
E15.5
E16.5
C
E16.5
D
E16.5
E17.5
E18.5
E
E16.5
E17.5
E18.5
F
E16.5
E17.5
E18.5
G
E17.5
H
E17.5
E18.5
I
E17.5
E18.5
J
E17.5
E18.5
K
E18.5
E19.5 / PD0
L
E18.5
E19.5 / PD0
M
E18.5
E19.5 / PD0
N
E18.5
E19.5 / PD0
PD1
O
E18.5
E19.5 / PD0
P
E19.5 / PD0
PD1

Figure EV8. Individual time-lapse quantification of photoconverted Mito-Dendra2 intensity.

Green Mito-Dendra2 in a germ cell within cultured gonads/ovaries was photoconverted to Red Mito-Dendra2, followed by 3D tracking and imaging at 5-min intervals. Absolute mean intensities of Red Mito-Dendra2 in photoconverted germ cells, along with one or two adjacent non-photoconverted germ cells, were measured and aligned by developmental time. Ex vivo time points were converted to their corresponding in vivo developmental times, as shown at the top of each plot. (**A**) Axes titles and legends. (**B–P**) Plots of quantified Red Mito-Dendra2 intensity. The samples used for imaging include: (**B**) E12.5 + 3 d gonad, (**C**) E12.5 + 4 d gonad, (**D**) E12.5 + 4 d gonad, (**E**) E14.5 + 2 d ovary, (**F**) E14.5 + 2 d ovary, (**G**) E12.5 + 5 d gonad, (**H**) E12.5 + 5 d gonad, (**I**) E12.5 + 5 d gonad, (**J**) E12.5 + 5 d gonad, (**K**) E12.5 + 6 d gonad, (**L**) E12.5 + 6 d gonad, (**M**) E12.5 + 6 d gonad, (**N**) E12.5 + 6 d gonad, (**O**) E12.5 + 6 d gonad, (**P**) E12.5 + 7 d gonad. See also Fig. 5D,E. Source data are available online for this figure.

 © The Author(s)

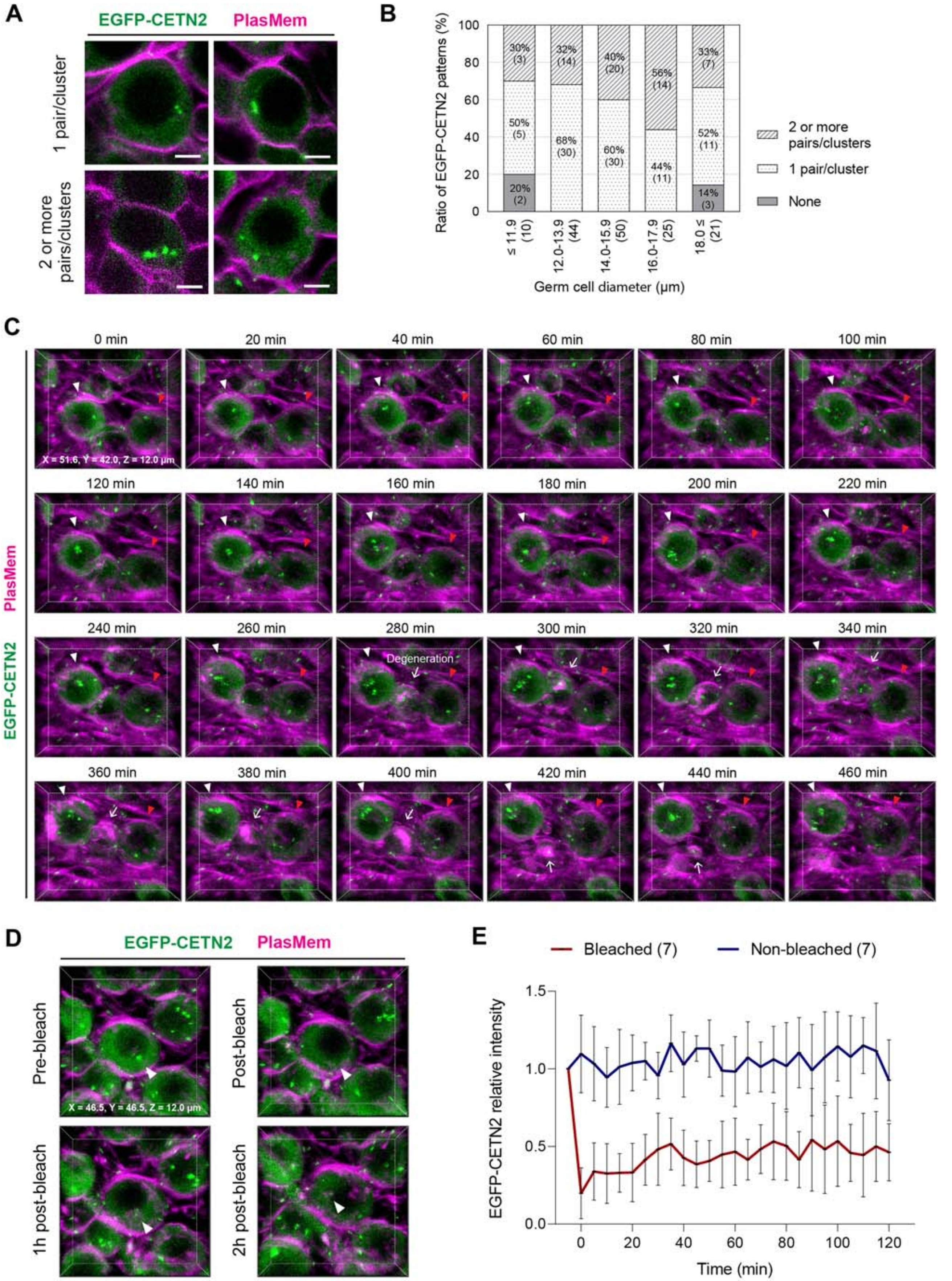
A
EGFP-CETN2 PlasMem
1 pair/cluster
2 or more pairs/clusters
B
Ratio of EGFP-CETN2 patterns (%)
Germ cell diameter (μm)
≤ 11.9 (10)
12.0-13.9 (44)
14.0-15.9 (50)
16.0-17.9 (25)
18.0 ≤ (21)
30% (3)
50% (5)
20% (2)
32% (14)
68% (30)
40% (20)
60% (30)
56% (14)
44% (11)
33% (7)
52% (11)
14% (3)
2 or more pairs/clusters
1 pair/cluster
None
C
0 min
20 min
40 min
60 min
80 min
100 min
120 min
140 min
160 min
180 min
200 min
220 min
240 min
260 min
280 min
300 min
320 min
340 min
360 min
380 min
400 min
420 min
440 min
460 min
X = 51.6, Y = 42.0, Z = 12.0 μm
Degeneration
PlasMem
EGFP-CETN2
D
EGFP-CETN2 PlasMem
Pre-bleach
Post-bleach
1h post-bleach
2h post-bleach
X = 46.5, Y = 46.5, Z = 12.0 μm
E
Bleached (7)
Non-bleached (7)
EGFP-CETN2 relative intensity
Time (min)

Figure EV9. Characterization of EGFP-CETN2 dynamics during oocyte formation.

(**A**) Patterns of EGFP-CETN2 signals in germ cells. Images were captured from E12.5 + 7 d (bottom left) and E12.5 + 9 d (top left, top right, and bottom right) gonads stained with PlasMem Bright Red. Merged images show EGFP-CETN2 (green) and anti-CETN2 (magenta). EGFP-CETN2 signal patterns include 1 pair (top left), 1 cluster (top right), and 2 or more pairs/clusters (bottom left and bottom right). Scale bar, 5 µm. (**B**) Distribution of EGFP-CETN2 patterns in germ cells by diameter. EGFP-CETN2 patterns in germ cells from E12.5 gonads cultured for 3 to 11 days were classified into three categories: None, 1 pair/cluster, and 2 or more pairs/clusters. Germ cell diameters were measured and grouped into five categories: ≤11.9 µm, 12.0– 13.9 µm, 14.0–15.9 µm, 16.0–17.9 µm, ≥18.0 µm. Numbers in brackets indicate germ cell counts. A total of 180 germ cells were analyzed, with 30 cells each from E12.5 + 3 d, + 5 d, + 7 d, + 9 d, and + 11 d gonads. See also Fig. 6A. (**C**) Representative live imaging of an E12.5 + 7 d gonad expressing EGFP-CETN2. The gonad was stained with PlasMem Bright Red and subjected to 3D time-lapse imaging every 10 min with z-sections at 1.5 µm intervals. Merged 3D images show EGFP-CETN2 (green) and PlasMem (magenta) signals displayed every 20 min in perspective. White and red arrowheads indicate tracked germ cells, respectively, while arrows denote germ cell degeneration. See also Movie EV11. (**D**, **E**) FRAP analysis of EGFP-CETN2 in germ cells. E12.5 + 7 d gonads expressing EGFP-CETN2 were stained with PlasMem Bright Red, followed by targeted photobleaching of EGFP-CETN2 signals in germ cells. (**D**) Representative 3D images of EGFP-CETN2 photobleaching. Merged images show EGFP-CETN2 (green) and PlasMem (magenta) signals, with arrowheads indicating the photobleached EGFP-CETN2 focus. (**E**) Time-lapse analysis of EGFP-CETN2 intensity in response to photobleaching. Mean EGFP-CETN2 intensity before photobleaching was normalized to a relative intensity of 1. The measurement area was manually defined using a circle with a diameter of 2 µm. The time point directly after photobleaching was set as 0 on the *x* axis. Bars represent mean values ± standard deviations. $N = 7$ photobleached germ cells and 7 non-photobleached germ cells. Source data are available online for this figure.

© The Author(s)

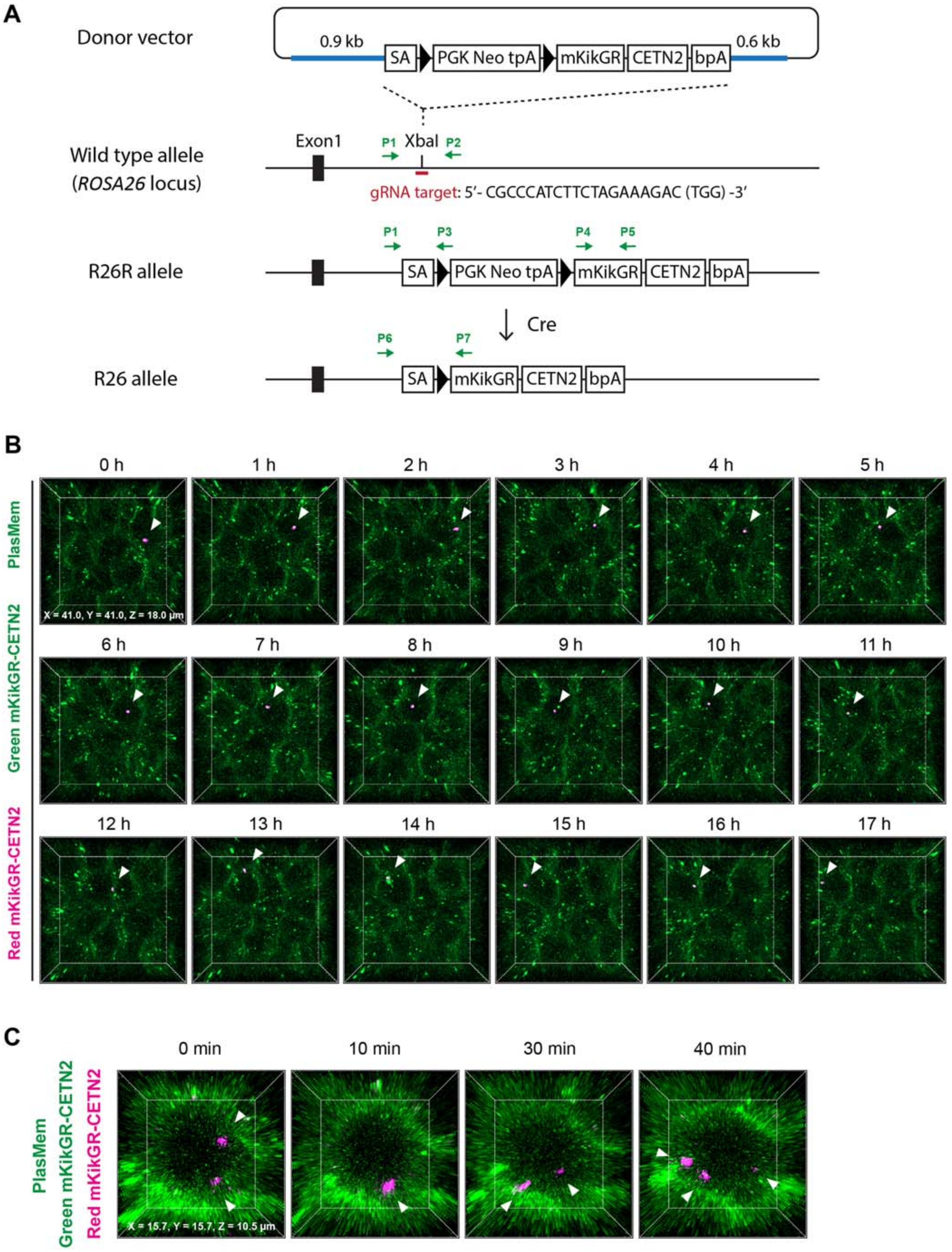
A
Donor vector
0.9 kb
SA
PGK Neo tpA
mKikGR
CETN2
bpA
0.6 kb
Exon1
Wild type allele
(ROSA26 locus)
P1
XbaI
P2
gRNA target: 5′- CGCCCATCTTCTAGAAAGAC (TGG) -3′
P1
P3
P4
P5
R26R allele
SA
PGK Neo tpA
mKikGR
CETN2
bpA
Cre
P6
P7
R26 allele
SA
mKikGR
CETN2
bpA
B
PlasMem
Green mKikGR-CETN2
Red mKikGR-CETN2
0 h
1 h
2 h
3 h
4 h
5 h
X = 41.0, Y = 41.0, Z = 18.0 µm
6 h
7 h
8 h
9 h
10 h
11 h
12 h
13 h
14 h
15 h
16 h
17 h
C
PlasMem
Green mKikGR-CETN2
Red mKikGR-CETN2
0 min
10 min
30 min
40 min
X = 15.7, Y = 15.7, Z = 10.5 µm

Figure EV10. Generation of mKikGR-CETN2 mice and characterization of mKikGR-CETN2 signals.

(**A**) Generation of mKikGR-CETN2 mice. The donor vector contains 5′ (0.9 kb) and 3′ (0.6 kb) homology arms (blue) targeting intron 1 of the ROSA26 locus, flanking an expression cassette. The vector was inserted into the genome of C57BL/6N zygotes using the CRISPR-Cas9 system with a guide RNA targeting the ROSA26 genomic locus. The resulting mice were crossed with Spo11-Cre mice to excise the stop sequences flanked by loxP sites. The black box represents exon 1 of the ROSA26 locus, and black triangles indicate loxP sequences. Green arrows indicate PCR primers. SA adenovirus splice acceptor sequence, PGK Neo neomycin resistance gene driven by the PGK1 promoter, tpA triple repeats of the SV40 polyadenylation sequence, mKikGR monomeric Kikume Green-Red, bpA bovine growth hormone polyadenylation sequence. (**B**) Representative time-lapse 3D Images of photoconverted Red mKikGR-CETN2 signals. An E12.5 + 5 d gonad expressing Green mKikGR-CETN2 was stained with PlasMem Bright Green, followed by photoconversion of Green mKikGR-CETN2 to Red mKikGR-CETN2. 3D time-lapse images of the photoconverted and neighboring germ cells were captured every 10 min with z-sections at 1.5 µm intervals. In the merged images, PlasMem and non-photoconverted Green mKikGR-CETN2 are shown in green, while photoconverted Red mKikGR-CETN2 is shown in magenta. Arrowheads indicate photoconverted Red mKikGR-CETN2 signals. See also Fig. 6F and Movie EV12. (**C**) Representative images of changes in mKikGR-CETN2 focal count. An E12.5 + 5 d gonad expressing mKikGR-CETN2 was stained with PlasMem Bright Green, followed by photoconversion of Green mKikGR-CETN2 to Red mKikGR-CETN2. 3D time-lapse images were captured every 10 min with z-sections at 1.5 µm intervals. Arrowheads indicate photoconverted Red mKikGR-CETN2, with focal counts varying over time. In merged images, PlasMem and non-photoconverted Green mKikGR-CETN2 are shown in green, while photoconverted Red mKikGR-CETN2 is shown in magenta.

 © The Author(s)

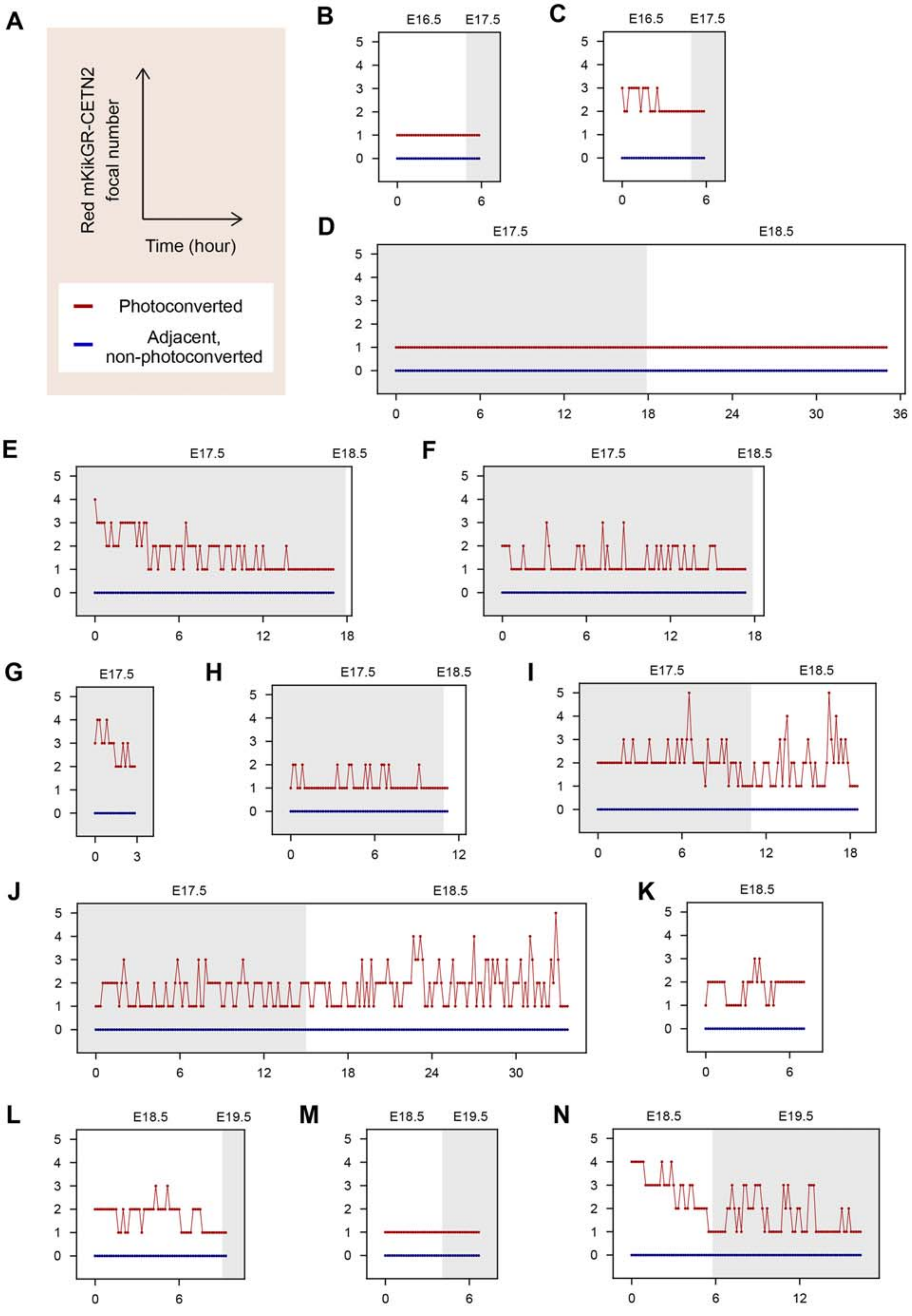
A
Red mKikGR-CETN2 focal number
Time (hour)
Photoconverted
Adjacent, non-photoconverted
B
E16.5
E17.5
C
E16.5
E17.5
D
E17.5
E18.5
E
E17.5
E18.5
F
E17.5
E18.5
G
E17.5
H
E17.5
E18.5
I
E17.5
E18.5
J
E17.5
E18.5
K
E18.5
L
E18.5
E19.5
M
E18.5
E19.5
N
E18.5
E19.5

Figure EV11. Individual time-lapse counts of photoconverted mKikGR-CETN2 foci.

Green mKikGR-CETN2 in a germ cell within cultured gonads was photoconverted to Red mKikGR-CETN2, followed by 3D imaging at 10-min intervals. The number of Red mKikGR-CETN2 foci in photoconverted germ cells and adjacent non-photoconverted germ cells was manually counted at each time point and aligned by developmental time. Ex vivo time points were converted to corresponding in vivo developmental times, as shown at the top of each plot. (**A**) Axes titles and legends. (**B**–**N**) Plots showing the number of Red mKikGR-CETN2 foci. The samples used for imaging include: (**B**) E12.5 + 4 d gonad, (**C**) E12.5 + 4 d gonad, (**D**) E12.5 + 5 d gonad, (**E**) E12.5 + 5 d gonad, (**F**) E12.5 + 5 d gonad, (**G**) E12.5 + 5 d gonad, (**H**) E12.5 + 5 d gonad, (**I**) E12.5 + 5 d gonad, (**J**) E12.5 + 5 d gonad, (**K**) E12.5 + 6 d gonad, (**L**) E12.5 + 6 d gonad, (**M**) E12.5 + 6 d gonad, and (**N**) E12.5 + 6 d gonad. See also Fig. 6G,H. Source data are available online for this figure.

 © The Author(s)
